# Validation of cardiac image-derived input functions for functional PET quantification

**DOI:** 10.1007/s00259-024-06716-8

**Published:** 2024-04-27

**Authors:** Murray Bruce Reed, Patricia Anna Handschuh, Clemens Schmidt, Matej Murgaš, David Gomola, Christian Milz, Sebastian Klug, Benjamin Eggerstorfer, Lisa Aichinger, Godber Mathis Godbersen, Lukas Nics, Tatjana Traub-Weidinger, Marcus Hacker, Rupert Lanzenberger, Andreas Hahn

**Affiliations:** 1 Department of Psychiatry and Psychotherapy, Medical University of Vienna, Vienna, Austria; 2 Comprehensive Center for Clinical Neurosciences and Mental Health (C3NMH), Medical University of Vienna, Vienna, Austria; 3 Department of Biomedical Imaging and Image-guided Therapy, Division of Nuclear Medicine, Medical University of Vienna, Vienna, Austria

**Keywords:** Image-derived input function (IDIF), Arterial input function (AIF), Functional positron emission tomography (fPET), [^18^F]2-fluoro-2-deoxy-D-glucose ([^18^F]FDG), 6-[^18^F]-fluoro-l-dopa (6-[^18^F]FDOPA)

## Abstract

**Purpose:**

Functional PET (fPET) is a novel technique for studying dynamic changes in brain metabolism and neurotransmitter signaling. Accurate quantification of fPET relies on measuring the arterial input function (AIF), traditionally achieved through invasive arterial blood sampling. While non-invasive image-derived input functions (IDIF) offer an alternative, they suffer from limited spatial resolution and field of view. To overcome these issues, we developed and validated a scan protocol for brain fPET utilizing cardiac IDIF, aiming to mitigate known IDIF limitations.

**Methods:**

Twenty healthy individuals underwent fPET/MR scans using [^18^F]FDG or 6-[^18^F]FDOPA, utilizing bed motion shuttling to capture cardiac IDIF and brain task-induced changes. Arterial and venous blood sampling was used to validate IDIFs. Participants performed a monetary incentive delay task. IDIFs from various blood pools and composites estimated from a linear fit over all IDIF blood pools (3VOI) and further supplemented with venous blood samples (3VOIVB) were compared to the AIF. Quantitative task-specific images from both tracers were compared to assess the performance of each input function to the gold standard.

**Results:**

For both radiotracer cohorts, moderate to high agreement (r: 0.60–0.89) between IDIFs and AIF for both radiotracer cohorts was observed, with further improvement (r: 0.87–0.93) for composite IDIFs (3VOI and 3VOIVB). Both methods showed equivalent quantitative values and high agreement (r: 0.975–0.998) with AIF-derived measurements.

**Conclusion:**

Our proposed protocol enables accurate non-invasive estimation of the input function with full quantification of task-specific changes, addressing the limitations of IDIF for brain imaging by sampling larger blood pools over the thorax. These advancements increase applicability to any PET scanner and clinical research setting by reducing experimental complexity and increasing patient comfort.

**Supplementary Information:**

The online version contains supplementary material available at 10.1007/s00259-024-06716-8.

## Introduction

Positron emission tomography (PET) imaging is a widely utilized method for visualizing and quantifying biological processes in vivo [1–4]. This includes the quantification of metabolic responses or neurotransmitter signaling during cognitive processing by the recently introduced framework of functional PET (fPET) [5, 6]. Using [^18^F]2-fluoro-2-deoxy-D-glucose ([^18^F]FDG), the approach has successfully identified task-relevant brain networks [3, 7] and revealed decoupling of glucose metabolism and hemodynamic signals [8, 9]. Furthermore, fPET has been utilized to quantify reward-specific changes in dopamine synthesis using 6-[^18^F]-fluoro-l-dopa (6-[^18^F]FDOPA) [10].

A pivotal aspect in understanding these physiological and pathological phenomena is the accurate quantification of PET data, which is achieved by dynamic acquisition and the characterization of spatio-temporal patterns of tracer kinetics. Compared to the standard uptake value (SUV), dynamic PET provides more robust outcome parameters [11–13]. However, full kinetic analysis at the voxel level is rarely carried out due to high noise, leading to less reliable parameter estimates and inconsistent models. Simpler graphical modeling methods aim to address these limitations, but in exchange, do not allow the separate estimation of each rate constant [11–13].

The above quantification techniques still rely on measuring the arterial input function (AIF), typically obtained through invasive blood sampling. However, this approach can be challenging and impractical, requires trained staff and additional medical effort, particularly in subpopulations where arterial access is compromised. A non-invasive alternative is the image-derived input function (IDIF), which extracts the input function from a blood pool within the PET images [14, 15]. In brain studies, the approach has not gained widespread application for several reasons. These include the limited spatial resolution of PET scanners, which leads to spill-over of activity from/to adjacent tissues, potentially affecting the accuracy and reliability of the IDIF, especially for small blood pools within the field of view (FOV) [16]. Particularly, the carotid arteries are prone to image noise and artifacts caused by patient motion, scanner instabilities, and low photon counts [16]. Additionally, carotid arteries are prone to partial volume effects due to their size being comparable to the spatial resolution of the scan. In contrast, larger blood pools in the thorax, such as the left ventricle and aorta, offer a more stable and accurate IDIF estimation [14]. However, these are not within the FOV when imaging the brain with conventional scanners.

As a solution, we employ a stop-and-go bed motion on a conventional scanner system, providing an alternating FOV between the thorax and brain, consequently enabling the acquisition of both the IDIF and the brain response to cognitive processing. This novel minimally invasive fPET scanning protocol enables quantifying metabolic changes or neurotransmitter synthesis using image-derived input functions (IDIFs) while overcoming former limitations. To allow generalizability, the approach is carried out for the quantification of glucose metabolism with [^18^F]FDG and dopamine synthesis with 6-[^18^F]FDOPA. IDIFs extracted from thoracic blood pools are validated with AIFs for both radioligands with respect to input function characteristics and final outcome parameters of net influx constants. Our main objective was to evaluate the validity of substituting the AIF with a non-invasive cardiac IDIF for brain fPET quantification, while still allowing posteriori frame reconstruction. Additionally, we aimed to investigate the impact of correcting for the plasma-to-whole-blood (pWB) ratio and the feasibility of conducting this correction using minimally-invasive venous samples as opposed to arterial samples in a step wise analysis. To achieve this, we compared the quantification obtained by gold standard AIF adjusted for pWB ratio using arterial samples, with (1) values quantified by IDIFs which were corrected for pWB using the venous samples and (2) without correcting for pWB.

## Materials and methods

### Participants

Twenty-one healthy individuals were recruited and underwent a single fPET/MRI examination using a Siemens Biography mMR scanner. Participants were injected with either [^18^F]FDG (age: 21 ± 1 years, 3/10 female) or 6-[^18^F]FDOPA (age: 24 ± 4 years, 4/10 female). One participant was excluded due to a failure of the automatic blood sampling system. All participants underwent a standard medical examination at the initial screening visit, which included blood tests, electrocardiography, neurological testing and the Structural Clinical Interview for DSM-IV performed by an experienced psychiatrist. Female participants also underwent a urine pregnancy test at the screening visit and before the PET/MRI scan. Exclusion criteria were current and previous (12 months) somatic, neurological or psychiatric disorders, current and previous substance abuse or psychotropic medication, current pregnancy or breast-feeding and previous study-related radiation exposure in the past 10 years. After detailed explanation of the study protocol, all participants gave written informed consent. Participants were insured and reimbursed for their participation. The study was registered in EudraCT (2019-004880-33) and approved by the Ethics Committee (ethics numbers: 2259/2017 and 2321/2019) of the Medical University of Vienna and procedures were carried out in accordance with the Declaration of Helsinki.

### Cognitive task

To examine reward and punishment processing, we employed a modified version of the well-established monetary incentive delay (MID) task. Participants were tasked with maximizing reward and minimizing loss by responding to stimuli within specific time limits. The task included 2 win and loss blocks (297s). Prior to the fPET scan, each participant’s individual reaction time was measured. Within each block, the probability of monetary gain and loss was manipulated by adjusting the reaction time limit by ± 50ms. This resulted in two blocks associated with higher potential monetary gains and two with higher potential monetary losses. During baseline phases, subjects were instructed to look at a crosshair, stay awake and let their minds wander. For a comprehensive description of the adapted MID implementation, please refer to the work by Hahn et al. [10].

### PET/MRI data acquisition

Synthesis of both tracers was performed each measurement day. Participants injected with 6-[^18^F]FDOPA received 150 mg Carbidopa and 400 mg Entacapone approximately 1 h prior to tracer application to block the peripheral metabolism of the radioligand by amino acid decarboxylase and catechol-O-methyl transferase [17, 18]. Both radioligands were administered simultaneously with fPET start using a bolus (510 kBq/kg/frame, 1 min) + constant infusion (40 kBq/kg/frame, 56 min) protocol, with a perfusion pump (Syramed µSP6000 with UniQUE MRI-shield, both Arcomed, Regensdorf, Switzerland) as described previously [3, 14].

**Fig. 1:**
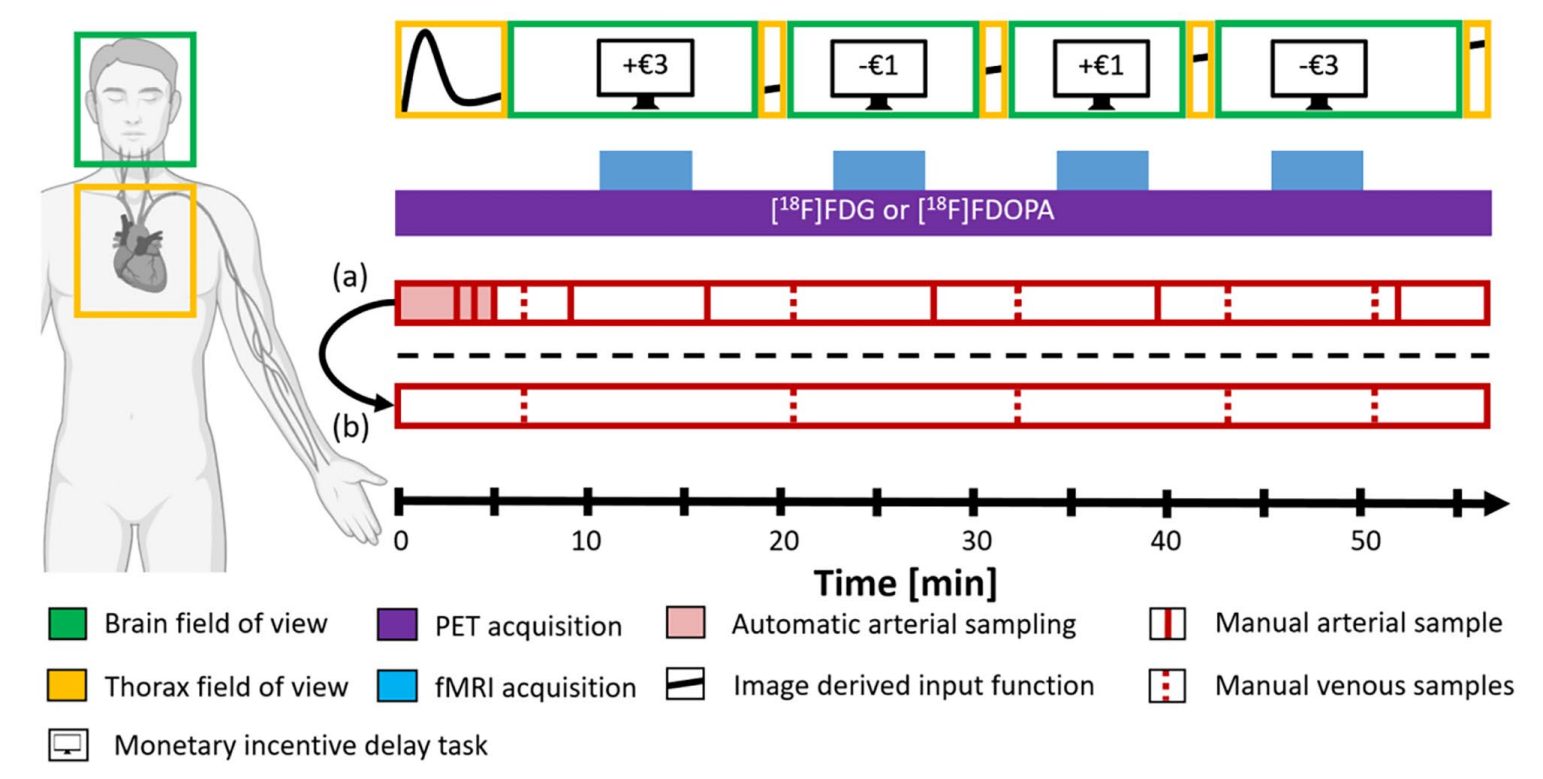
Graphical overview of the measurement protocol. For each participant, the PET scan starts with the PET-FOV placed over the thorax (orange box) for 5:30 min to acquire the initial peak of the tracer. During this time three manual arterial blood samples are taken at minutes 3, 4 and 5 (red line). Afterwards, the bed position is moved to the brain (green box) and fPET acquisition is started while the participant views a cross and lets their thoughts wander. At minute 11 the first MID task block begins parallel to the start of the fMRI sequence. Before and after task performance, both manual arterial and venous samples are taken. Following the end of the brain block the bed automatically moves to the thorax to acquire further data points for the IDIF in a stop-and-go bed motion. This process is repeated multiple times to provide robust estimates for both the IDIF and fPET task metrics. (**a**) depicts the protocol used to validate the IDIFs. (**b**) indicates the final simplified protocol to calculate fully quantified data

fPET data were collected in list-mode using a stop-and-go bed movement strategy to alternate between brain and thorax regions with a spatial resolution of (x, y, z) 2.09 × 2.09 × 2.03 mm and a matrix size of 344 × 344 × 127 voxels. The fPET scan started over the thorax, allowing for the determination of image-derived input functions (IDIF) from the left ventricle, ascending, and descending aorta. After 6 min, the bed moved to the brain field of view to acquire baseline and MID task data (4 × 5 min) in a block design. After each task block, the bed returned to the thorax to acquire additional IDIF data points (4 × 30s). This process was repeated multiple times to obtain reliable IDIF, baseline, and task data (Fig. 1).

Structural MRI of the brain was acquired before fPET using a T1-weighted MPRAGE sequence (TE/TR = 4.21/2200 ms, TI = 900 ms, flip angle = 9°, matrix size = 240 × 256, 160 slices, voxel size = 1 mm isotropic, TA = 7:41 min), which was used for spatial normalization. Whereas, the thorax was acquired using a T1-weighted STARVIBE sequence (TE/TR = 1.44/3050 ms, flip angle = 5°, matrix size = 320 × 320, 208 slices, voxel size = 1.19 × 1.19 × 1.2 mm, TA = 5:33 min) and used for IDIF localization.

MID functional data were acquired using an EPI sequence (TE/TR = 30/2000 ms, flip angle = 90°, matrix size = 80 × 80, 34 slices, voxel size = 2.5 × 2.5 × 2.5 mm + 0.825 mm gap).

### Blood sampling and input function construction

For the first 5.5 minutes, blood was automatically sampled from the radial artery (Twilite II system; Swisstrace) and corrected for external delay. Furthermore, additional manual samples were obtained at 3, 4 and 5 minutes after tracer administration. Thereafter, during baseline phases, both manual arterial (9, 16.5, 28, 39.5 and 52 min) and venous blood (7, 20, 31.5, 43, 51 min) samples were drawn. After each measurement, plasma was separated from whole blood, and the activity of both was measured using a γ-counter (Wizward2, 3”, Perkin Elmer), which was cross-calibrated to the PET/MR scanner.

The AIF was constructed by combining the activities obtained from the automatically and manually collected samples. For IDIFs, three fixed-size volumes of interest (VOIs) were manually placed in the left ventricle, ascending and descending thoracic aorta using both the mean PET image and structural thorax T1 as reference. For the ascending and descending aorta, a cylindrical VOI with a diameter of 3.13 mm and a length of 12.54 mm was used. The left ventricle VOI was defined as a spherical VOI with a diameter of 9.9 mm. Moreover, we increased the VOI size for the aorta to 14.63 mm and for the ventricle to 12.54 mm in accordance with [19] to assess robustness. The mean activity within each VOI was extracted for each time point, representing the IDIFs. Intended positioning for each IDIF can be seen in Supplementary Fig. 1a. Representative single-subject summed [^18^F]FDG and 6-[^18^F]FDOPA PET thorax images, illustrating the discernible signal, are available in Supplementary Fig. 1b and c.

In addition, two composite IDIFs were created as follows. The initial peak was extracted from the left ventricle’s initial time course during the first thorax bed position, which has been shown to be most accurate [14] but as this does not affect linear quantification, other pools may also be used, i.e. descending aorta in patient cohorts that exhibit high movement during measurements [14]. The succeeding tail of the composite input function was created by fitting a 1st order polynomial function (linear function) using each of the ascending, descending aorta and left ventricle IDIF’s sampling time points as an inputs, referred to as 3VOI. These time points were sampled over the thorax at 5, 18.5, 30, 41.5 and 55.5 min after infusion start. The resulting fit was performed using 15 data points per participant. This was done to increase the robustness against possible movement-induced inaccuracies in the IDIF extraction of a single VOI. The second composite IDIF was estimated by additionally including all venous samples available for each participant to 3VOI’s fit (see sample timings above), which aims to further improve the accuracy of the linear fit, henceforth named 3VOIVB. Both arterial and venous samples, as well as all IFs were temporally aligned with the PET frame acquisition. This was achieved by linearly interpolating the data to the brain PET frames, which were reconstructed to 30s intervals.

In a further step, the resulting IDIFs were multiplied with the pWB ratio from their manual venous blood samples which was estimated by averaging the samples for [^18^F]FDG [3, 5] and applying a linear fit for 6-[^18^F]FDOPA data [10]. The pWB ratio for the AIF was corrected for using arterial blood samples. As the intake of carbidopa and entacapone [20] combined with the bolus + constant infusion (B + I) radioligand administration [10] substantially reduces the amount of radioactive metabolites of 6-[^18^F]FDOPA, a literature-based correction was used (Supplementary Fig. 3) [20]. The fraction of 6-FDA was fitted with a single exponential function and converted to match the B + I protocol. Additionally, we conducted simulations to investigate the potential biasing effects of increasing the 6-FDA metabolite by 10%, 20%, 30%, and 40% on the quantification process.

### Processing data

Each list-mode PET block was reconstructed using the ordinary Poisson ordered subset expectation maximization algorithm (3 iterations, 21 subsets; see supplement for framing and correction methods). The thorax and brain frames were concatenated separately and decay corrected to the start of the measurement. The first thorax block was binned (20 × 5s, 8 × 10s, 5 × 30s) to accurately map the initial tracer kinetics. The remaining brain and thorax blocks were binned into 30s frames. Attenuation and scatter correction were performed for brain blocks using a pseudo-CT approach based on the structural T1 [21], while the thorax blocks were corrected using the DIXON MRAC with a CAIPIRINHA sampling pattern [22]. The distance between the head and thorax field of view was set to 335 mm for all participants.

The thorax and brain frames were concatenated separately and decay corrected to the start of the measurement. Brain data were preprocessed using SPM12 (Wellcome Trust Centre for Neuroimaging), as described previously [7]. Briefly, fPET data were corrected for head motion (quality = best, registered to mean) and coregistered to the structural T1. The structural MRI was spatially normalized to MNI space, and the transformation matrix was applied to the coregistered fPET images. Images were smoothed with an 8 mm Gaussian kernel, masked to include only gray matter voxels and a low-pass filter was applied with the cutoff frequency set to 2.5 min.

A general linear model was utilized to extract task effects from baseline metabolism. The model included task regressors for win- and lose-blocks. Additionally, principal components of the six motion parameters explaining more than 90% of variance were added as motion regressors. The baseline was defined as average across all grey matter voxels, excluding those active during fMRI task performance (contrast success > failure, *p* <  0.011 uncorrected) and those identified in a meta-analysis of the MID task (contrasts reward/loss anticipation and reward outcome) [10]. One frame before and after bed movement was deweighted to 0.5 to reduce potential effects induced by the bed movement. Moreover, the infusion start was denoted within all task and nuisance regressors were denoted with a (0, 0) point.

The Gjedde-Patlak plot was used to estimate voxel-wise CMRGlu and net influx constant Ki for [^18^F]FDG and 6-[^18^F]FDOPA, respectively. The slope was fitted from t*=20 min after infusion start.

### Statistical analysis

**Fig. 2:**
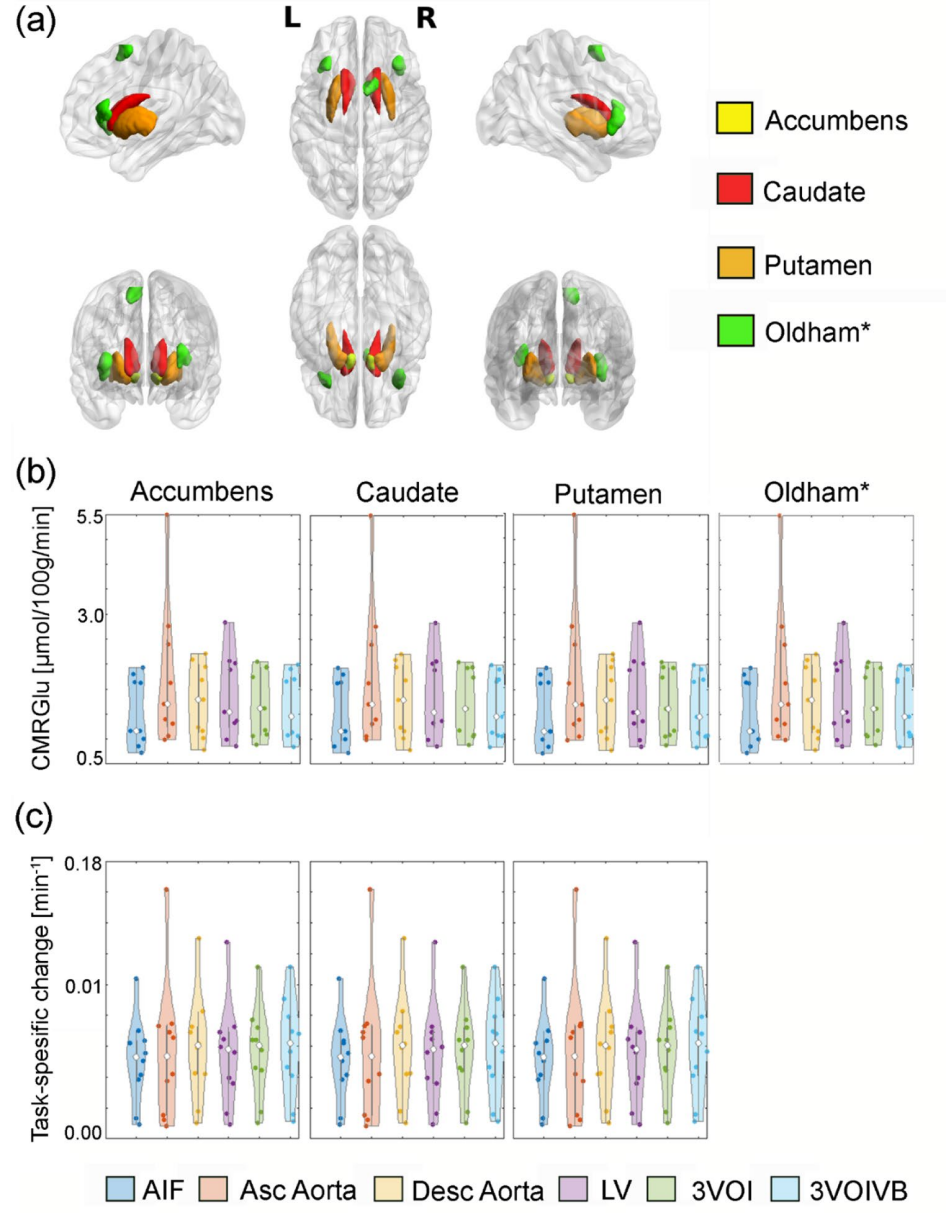
(**a**) depicts a graphical overview of all region of interest used in both [^18^F]FDG and 6-[^18^F]FDOPA cohorts. The green (Oldham) cortical regions were extracted from the conjunction analysis of reward and loss anticipation from Oldham et al. [23]. The Oldham regions of interest were not used for the 6-[^18^F]FDOPA cohort. Created using BrainNet Viewer [38]. (**b**) shows different cerebral metabolic rate of glucose values extracted from each of the four regions and input functions from the [^18^F]FDG cohort, whereas (**c**) displays task-specific changes for the 6-[^18^F]FDOPA cohort after correction for plasma to whole blood ratio

To evaluate the similarity between the IDIFs and the gold standard AIF, we conducted linear regression analysis and computed Pearson correlation coefficients for both the area under the curve and peak values. Furthermore, regional values of CMRGlu and Ki were extracted using the Harvard Oxford subcortical atlas and cortical regions from the Oldham meta-analysis (reward anticipation and loss anticipation, Fig. 2) [23]. We then assessed the linear relationship between outcome parameters obtained from IDIFs and AIF (i.e., CMRGlu values from [^18^F]FDG and 6-[^18^F]FDOPA Ki) for both win and loss conditions using Pearson’s correlation coefficient and linear regression analysis.

Furthermore, to determine statistical equivalence between the gold standard (AIF corrected for arterial pWB), to IDIFs (corrected for venous pWB), we employed the two one-sided t-tests (TOST) to determine if regional Ki values quantified by AIF and IDIFs were equivalent i.e. statistically the same [24]. Subsequently, we tested to see whether a venous correction for pWB ratio was necessary by testing the equivalency between AIF corrected for arterial pWB and IDIFs without pWB correction.

Finally, we tested for potential bias between venous and arterial blood samples using a paired t-test on mean values for the [^18^F]FDG cohort. For the 6-[^18^F]FDOPA cohort, each venous blood sample was interpolated to match the time of the arterial sample, and individual comparisons were made using a paired t-test. The significance level was *p* < 0.05 for all tests.

## Results

### Comparison of input functions

**Fig. 3:**
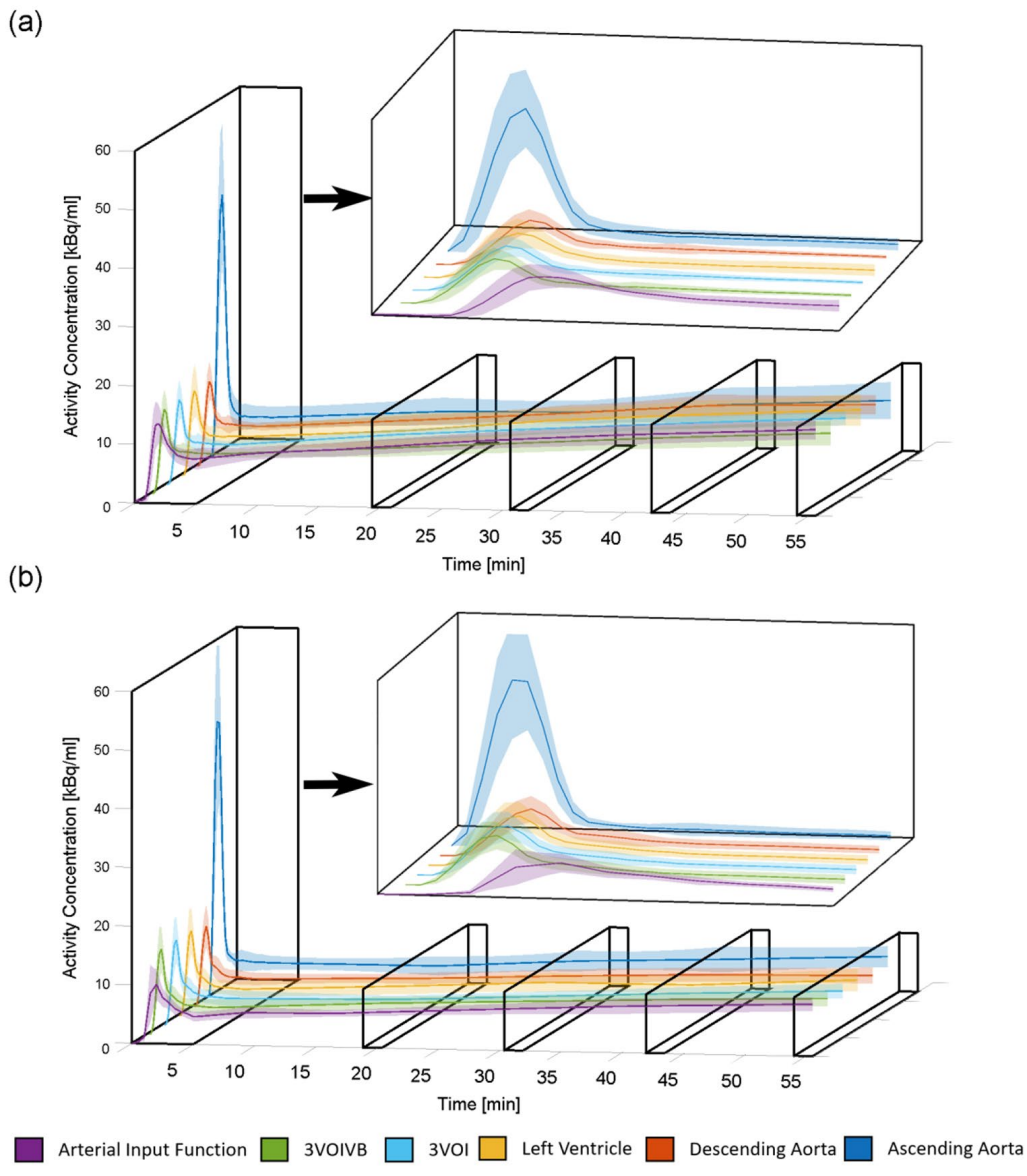
Graphical overview of all input functions. The mean and standard deviation of each input function’s time course for the [^18^F]FDG (**a**) and 6-[^18^F]FDOPA cohort (**b**). The top right inlay represents the first 5 min of the entire time course. The boxes indicate the times when the FOV was shuttled from the brain to the thorax to record the image-derived input function. All input functions display a similar peak. The arterial input function (AIF) peaks later than all image-derived input functions. Finally, both the 3VOI and 3VOIVB display the highest similarity to the AIF

Figure 3 provides a visual representation of the time course of each input function for both [^18^F]FDG (a) and 6-[^18^F]FDOPA (b) cohorts. The peak values observed in the ascending aorta IDIF were significantly higher than those of all other IDIFs and the AIF (*p* <  0.01). However, when using the IDIFs extracted via the larger VOIs no significant differences in peak values between the AIF and the other IDIFs (all *p* > 0.4) remained, see Supplementary Fig. 1, Supplementary Tables 1 and 2. This difference is visually depicted in Fig. 3, for the smaller VOIs and Supplementary Fig. 2, for the larger VOIs. The AIF exhibited a later peak (mean ± SD: 110 ± 13s) compared to the ascending aorta (mean ± SD: 56 ± 5s), descending aorta (mean ± SD: 72 ± 5s), left ventricle, 3VOI, and 3VOIVB (mean ± SD: 71 ± 6s).

**Fig. 4:**
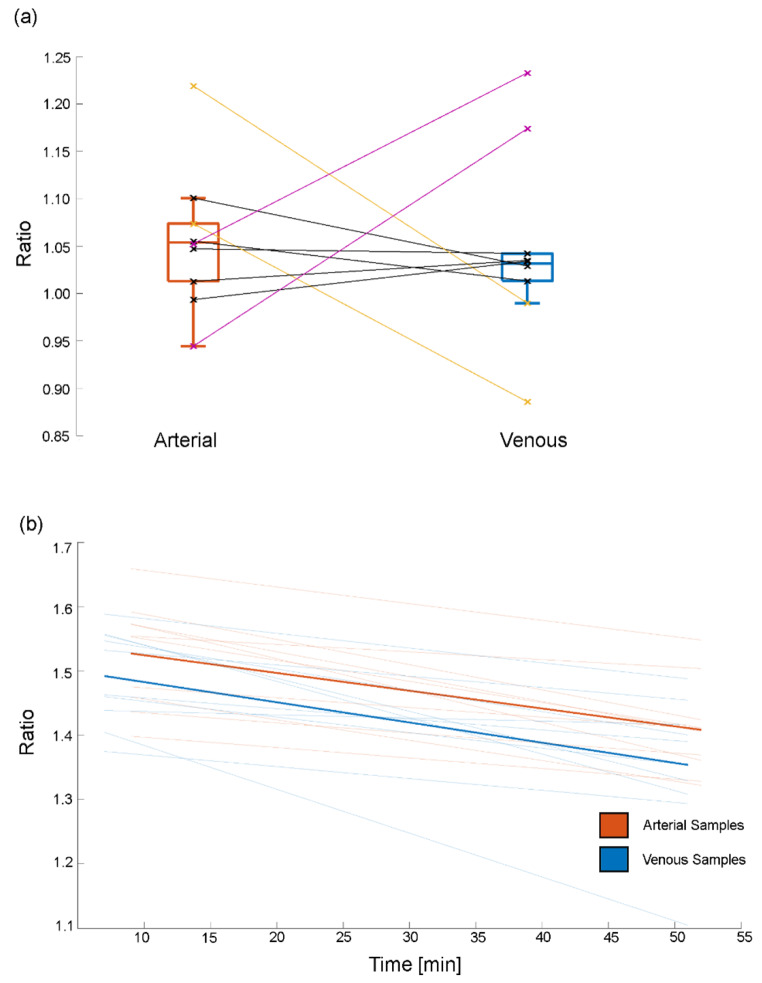
Comparison of arterial and venous plasma to whole-blood ratios for [^18^F]FDG (**a**) and 6-[^18^F]FDOPA (**b**). While (**a**) shows no significant difference between arterial and venous samples, some outliers (inaccurately measured blood sample(s)) were present. Each point and line represent the average venous and arterial blood level per participant. The yellow outliers’ late venous samples were not collected resulting in an underestimation. In comparison, purple initial venous samples were not collected, resulting in an overestimation. All black lines represent participants were all blood samples were taken. (**b**) Shows the linear fit averaged over all participants of both arterial (bold orange) and venous (bold blue) samples and also individual fits. In the case of 6-[^18^F]FDOPA, a linear underestimation from the venous samples can be seen (blue) when compared to arterial (orange)

Both cohorts demonstrated comparable degrees of correlations and regression analysis between IDIFs and AIF (Table 1). Generally, the left ventricle IDIF showed the highest similarity with the AIF (AUC(r) = 0.79–0.84). The match with the AIF increased for the composite IDIF (3VOI(r) = 0.9–0.92) and exhibited a similar match when combined with the venous blood samples (3VOIVB(r) = 0.88). No significant difference in [^18^F]FDG pWB ratio was found between arterial and venous samples (*p* = 0.82, Fig. 4a). However, a significant underestimation in venous 6-[^18^F]FDOPA concentration was observed at multiple time points (*p* < 0.02, Fig. 4b).

**Table 1:** Comparison of each image-derived input function to the gold standard, arterial input function for both [^18^F]FDG and 6-[^18^F]FDOPA tracers. Both the area under the curve and peak values are compared using the Pearson correlation as well as regression analysis. Bold values indicate the highest correlations per parameter and tracer

VOI	Metric	[^18^F]FDG			6-[^18^F]FDOPA		
		r	Slope	Intercept	r	Slope	Intercept
Ascending Aorta	AUC	0.82	0.87	-724.506	0.83	0.73	9158.662
	Peak	0.61	2.35	9.225	0.86	4.61	-3.462
Descending Aorta	AUC	0.64	0.54	11233.711	0.83	0.91	-152.674
	Peak	0.79	0.66	5.131	0.66	1.02	-1.647
Left Ventricle	AUC	0.83	0.95	-3477.269	0.89	1.38	-10552.293
	Peak	0.60	0.51	7.608	0.82	1.38	-1.891
3VOIF	AUC	0.91	0.75	4593.352	0.87	1.10	-3488.225
	Peak	0.60	0.51	7.608	0.82	1.38	-1.891
3VOIFVB	AUC	0.92	0.84	2123.264	0.93	1.01	-3008.017
	Peak	0.60	0.51	7.608	0.82	1.38	-1.891

### Input function effects on quantified values

The highest correlations of regional CMRGlu and Ki values were found between the AIF and both 3VOI and 3VOIVB IDIFs (*r* = 0.957–0.998, Table 2). Accordingly, TOST tests revealed that CMRGlu and Ki values corrected for venous pWB, obtained using the 3VOI and 3VOIVB IDIFs were equivalent to those quantified using the AIF which was also corrected for arterial pWB (all *p* < 0.025, Table 3) over all regions (Fig. 2b, c). However, quantified task-induced values derived from the singular thoracic blood pools were not equivalent to the AIF-based results (*p* > 0.05, Table 3) for all regions. Similarly, quantified task-induced values derived from the larger VOIs (Supplementary Table 3), were not equivalent to the AIF over all regions. Moreover, TOST tests on IDIFs not corrected for pWB were not equivalent (*p* > 0.05) for all regions.

**Table 2:** Pearson correlation and regression analysis of task-induced [^18^F]FDG CMRGlu and 6-[^18^F]FDOPA Ki over all participants between image-derived and arterial input functions. *Cortical regions extracted from the Oldham meta-analysis of the monetary incentive delay task [23]. Bold values represent the best correlation for each tracer and region

Tracer			[^18^F]FDG					6-[^18^F]FDOPA				
Input Function			Asc Aorta	Desc Aorta	LV	3VOI	3VOIVB	Asc Aorta	Desc Aorta	LV	3VOI	3VOIVB
Brain Regions	Accumbens	r	0.955	0.984	0.988	0.993	**0.995**	0.907	0.984	0.983	**0.988**	0.985
		Slope	2.15	1.15	1.40	1.06	**1.02**	1.45	1.21	1.16	**1.06**	1.11
		Intercept	-1.72	0.10	-0.43	0.23	**0.20**	< 0.01	< 0.01	< 0.01	**< 0.01**	< 0.01
	Caudate	r	0.903	0.983	0.980	0.992	**0.992**	0.879	0.969	0.973	**0.975**	0.957
		Slope	1.48	1.12	1.21	1.08	**1.03**	1.21	1.25	1.17	**1.15**	1.11
		Intercept	-0.26	0.03	-0.05	0.05	**0.05**	< 0.01	< 0.01	< 0.01	**< 0.01**	< 0.01
	Putamen	r	0.938	0.984	0.987	0.988	**0.987**	0.841	0.971	0.968	**0.974**	0.965
		Slope	2.25	1.18	1.45	1.08	**1.04**	1.44	1.16	1.12	**1.05**	1.09
		Intercept	-0.44	-0.02	-0.13	0.03	**0.02**	< 0.01	< 0.01	< 0.01	**< 0.01**	< 0.01
	Oldham*	r	0.989	0.989	0.992	0.997	**0.998**	-	-	-	-	-
		Slope	1.36	1.07	1.14	1.07	**1.06**	-	-	-	-	-
		Intercept	-0.62	< 0.01	-0.17	0.09	**0.06**	-	-	-	-	-

**Table 3:** Overview of equivalency test results estimated for regional net influx rates (Ki) quantified using each image derived-input function and arterial input function (significance indicates equivalence). Cohen’s d represents the standardized mean difference between the AIF and each IDIF. *Cortical regions extracted from the Oldham meta-analysis of the monetary incentive delay task [23]

Tracer	Input Function	Cohen’s d	Brain Regions							
			Caudate		Putamen		Accumbens		Oldham*	
			*p*-value(1)	*p*-value(2)	*p*-value(1)	*p*-value(2)	*p*-value(1)	*p*-value(2)	*p*-value(1)	*p*-value(2)
[18 F]FDG	Asc Aorta	0.19	0.32	0.42	0.19	< 0.01	0.17	< 0.01	0.29	0.15
	Desc Aorta	0.18	0.12	0.06	0.02	< 0.01	< 0.01	< 0.01	0.12	0.06
	Left Ventricle	0.11	0.43	0.39	0.04	< 0.01	0.01	< 0.01	0.17	0.09
	3VOIF	0.33	**0.03**	**0.01**	**< 0.01**	**< 0.01**	**< 0.01**	**< 0.01**	**0.03**	**0.01**
	3VOIFVB	0.22	**0.02**	**0.01**	**< 0.01**	**< 0.01**	**< 0.01**	**< 0.01**	**0.05**	**0.03**
6-[18 F]FDOPA	Asc Aorta	0.55	0.36	0.25	0.19	0.03	0.25	0.07	N/A	N/A
	Desc Aorta	0.54	< 0.01	< 0.01	< 0.01	< 0.01	< 0.01	< 0.01	N/A	N/A
	Left Ventricle	0.59	0.05	0.01	0.03	< 0.01	0.03	< 0.01	N/A	N/A
	3VOIF	0.69	**0.01**	**< 0.01**	**< 0.01**	**< 0.01**	**< 0.01**	**< 0.01**	**N/A**	**N/A**
	3VOIFVB	0.05	**< 0.01**	**< 0.01**	**< 0.01**	**< 0.01**	**< 0.01**	**< 0.01**	**N/A**	**N/A**

Additionally, the magnitude of CMRGlu and Ki values induced by the task in both win and loss conditions were consistent with those reported in previous literature [5, 10].

Simulations were conducted to assess the impact of increased 6-FDA metabolite fractions on Ki values. The results showed subtle changes, with a 10% group increase in 6-FDA resulting in a 0.85% increase in task-specific Ki values across ROIs. Subsequent simulations with 20%, 30%, and 40% increments demonstrated corresponding increases of 2.2%, 5.2%, and 8.4%, respectively. According to Ishikawa et al., a variation of up to 30% is plausible [20].

## Discussion

**Fig. 5:**
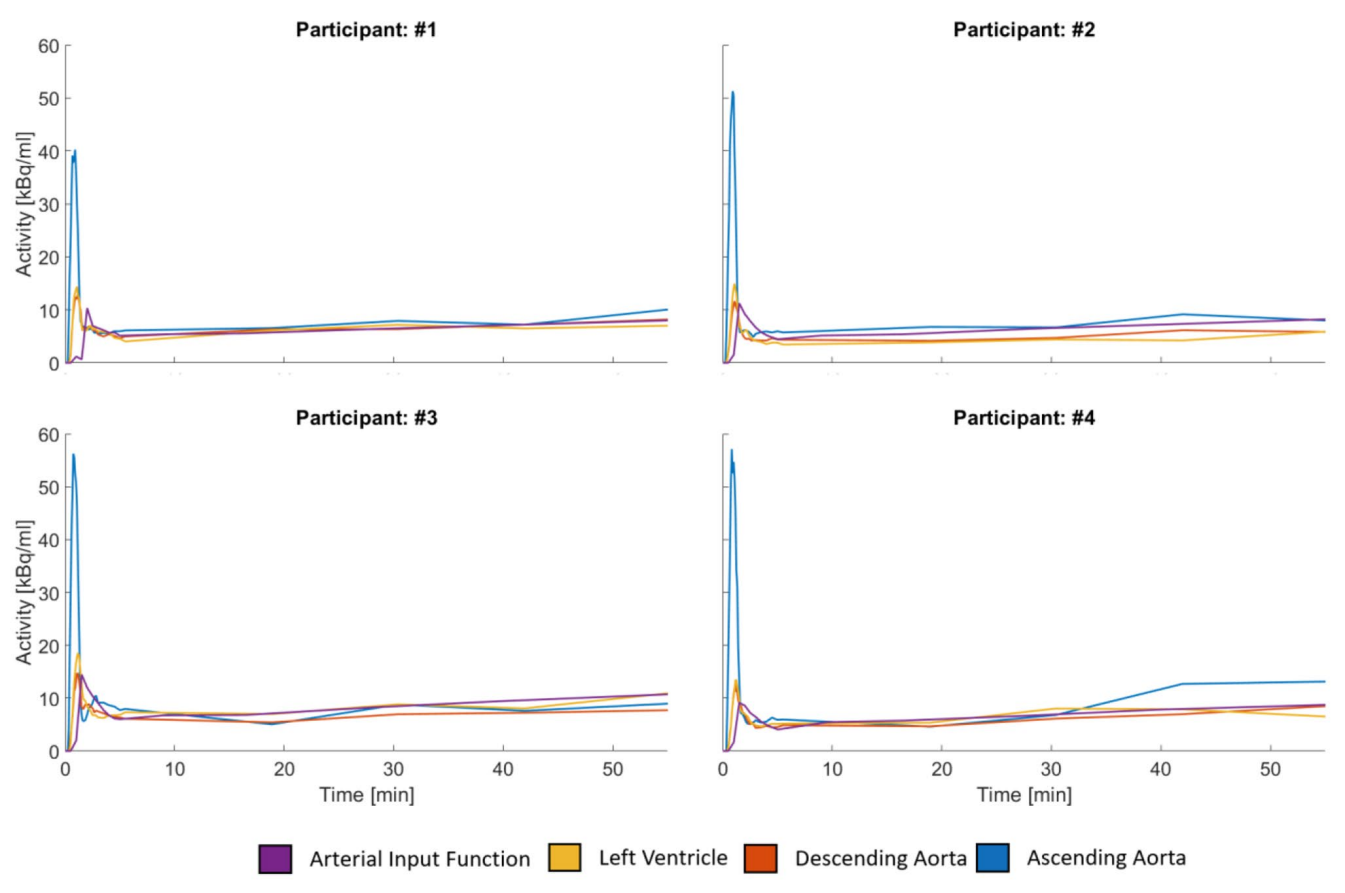
Individual input function time courses of four participants. The shape of the image derived input functions extracted from the left ventricle, ascending and descending aorta varied from the arterial input function, which is more visible during the later stages of the measurement. Participants 1 and 2 where from the [^18^F]FDG cohort, while participants 3 and 4 where from the 6-[^18^F]FDOPA cohort

The aim of the study was to compare cardiac IDIFs to the gold standard AIF and assess the validity of derived quantitative values in an fPET framework for multiple tracers. The results demonstrated a variable agreement of the different IDIFs compared to the AIF. While the IDIFs extracted from a single blood pool showed a moderate to high match in peak values and AUC, the shape of the input function varied at certain time points (Fig. 5), which influenced the accuracy of task quantification. Both composite IDIFs without (3VOI) and with venous blood samples (3VOIVB) exhibited excellent agreement for input function and outcome parameters, with regional quantified values being equivalent to those derived from the AIF. Moreover, the performance of the composite IDIFs was statistically equivalent to the AIF for both radiotracers [^18^F]FDG and 6-[^18^F]FDOPA, indicating the generalizability of the approach when correcting for pWB. However, IDIFs not corrected for pWB were not equivalent to the gold standard of AIF. By providing a simplified protocol that does not require arterial blood samples (Fig. 1b), these advancements increase applicability to any PET scanner and also clinical research setting by reducing experimental complexity and increasing patient comfort.

Next-generation PET/CT scanners exhibit improved spatial and temporal resolution, along with a broader field of view, improving blood pool selection, tissue differentiation and subsequent IDIF extraction [14, 25]. Despite these improvements, extracting IDIF from conventional arterial blood pools remains challenging and require additional acquisitions e.g. time-of-flight MR angiography and navigators for carotid segmentation and movement detection [26]. Small vessels such as the carotid, brachial, and femoral arteries are still affected by dispersion and partial-volume effects due to their size [27]. Even with an effective sensitivity increase of up to 40 times compared to previous generations (e.g., Biograph mCT), the Biograph Quadra encounters challenges in accurately extracting an IDIF from the internal carotid [28]. It has been demonstrated that these highly sensitive scanners face difficulties in IDIF extraction from small blood pools, limiting the accurate estimation of kinetic microparameters while allowing for the estimation of Ki [28]. This underscores the necessity of the implementation of alternative IDIF extraction methods from larger blood pools, such as the one utilized in our study, or considering techniques like CBM or employing whole-body scanners. Moreover, it highlights the imperative requirement of a PET scanner with high spatial resolution for IDIF extraction via internal carotids. Utilizing larger blood pools in the thorax can help alleviate these problems and improve accuracy [25]. To this end, manufacturers are introducing vendor software with their next-generation scanners, such as GE, United Imaging [14] and Siemens [29], leveraging continuous bed motion (CBM) for whole-body imaging acquisition and automated IDIF extraction for Ki estimation. However, these solutions have limitations. Notably, they entail a predetermined frame length, adjustable after the acquisition is started due to CBM, and are limited to [^18^F]FDG. Additionally, the inability to continuously acquire dynamic PET data over specific fields of view, such as the brain, poses a constraint, particularly for quantification of stimulation-induced changes as with fPET. Moreover, the automatic IDIF extraction in existing software packages may yield inaccurate results due to the misplacement of VOIs [14]. Our method provides a flexible and tailored acquisition for both IDIF and fPET data acquisition timing, along with accurate IDIF extraction for multiple tracers. Our results further highlight that an accurate IDIF extraction from thoracic blood pools can also be performed on widely available PET/MR or PET/CT scanners, not limited to next-generation scanners with time-of-flight or vendor specific software packages utilizing CBM for whole-body data acquisition. By modelling the IDIF time course using a composite of multiple blood pools even further increased the agreement with the AIF gold standard.

While larger thoracic blood pools provide more accurate IDIF estimation, their acquisition with a small FOV scanner (e.g., with stop-and-go as well as CBM) limits quantification to graphical approaches, which only allows for the estimation of the net influx constant. These graphical methods do not require that the shape of the initial part of the input function be precisely estimated, as they mainly rely on the area under the curve. Thus, graphical methods are less affected by IDIF errors and, in this context provide, more robust quantification than compartmental modeling [16]. Despite their potential, graphical methods have been shown to be potentially susceptible to bias [30], primarily influenced by the accuracy of estimating the later segments of the input functions. Incorporating blood samples for scaling purposes can greatly improve the accuracy of the IDIF curves’ tail [30]. Our results also suggest that using venous samples improves both IDIF shape and quantification accuracy but to a lesser degree than previously reported, indicating that the benefit depends on the radiotracer. In line, a recent study, employing a highly sensitive next-generation scanner, has demonstrated that that accuracy of IDIF from larger blood pools varies depending on the tracer applied, necessitating validation for each tracer [31]. Our study contributes to these efforts by corroborating their conclusions regarding the highly accurate IDIF extraction of [^18^F]FDG on conventional PET scanners and extends it to include 6-[^18^F]FDOPA.

The utilization of stop-and-go bed movement has been employed in previous studies in the framework of whole-body acquisition similar to CMB [32, 33]. This approach circumvents the restrictions a small scanner FOV pose to acquire whole-body images, with the drawback of having long frames, which are not ideal for fPET. The shuttle-mode between the brain and thorax has yet to be explored in detail. This approach allows for flexible acquisition of both fPET brain and thorax IDIF acquisition in list-mode offering the advantage of adaptable framing. By dynamically shifting the bed between the brain and thorax, this technique enhances the temporal and spatial resolution of imaging, providing a versatile and efficient means of obtaining comprehensive data for quantitative analysis in neuroimaging studies.

The strong performance of the IDIFs for both [^18^F]FDG and 6-[^18^F]FDOPA suggests that the proposed approach is generalizable, at least for radioligands with (partly) irreversible kinetics and subsequent quantification with the Patlak plot. Still, we propose that this may also be successfully extended to the quantification of certain reversible radioligands with the Logan plot. Although this graphical approach requires the integral of both blood and tissue activity [34], radioligands with slow tissue kinetics may not require full sampling of the initial part of the time activity curve. On the other hand, most reversible radioligands require the determination of radioactive metabolites, usually from blood samples. Here, it is important to acknowledge that arterial tracer kinetics may differ from venous ones [35, 36]. Substitution can only be done if the venous samples are obtained during a period of transient equilibrium. However, the time required to achieve this equilibrium varies for each tracer. This also applies to irreversible radioligands with respect to pWB ratio, e.g., an arteriovenous equilibrium for [^18^F]FDG is reached approximately 10 to 15 min after injection [37]. This can also be seen in our [^18^F]FDG cohort data, where we found no significant differences between venous and arterial samples. While our 6-[^18^F]FDOPA blood data also seemed to reach arteriovenous equilibrium around the same time window as [^18^F]FDG, there was a constant underestimation in the venous samples. However, this can be corrected for by implementing an additive factor for quantification. Of note, the underestimation in venous samples had no significant effect on the final outcome parameters. Here, the advent of long-axial FOV PET/CT scanners, which allow simultaneous recording of the brain response, thoracic IDIF and further organs involved in metabolism, holds promise for a non-invasive full compartmental modeling approach, where rate constants can be estimated without blood sampling.

While our non-invasive IDIF method eliminates the need for blood samples and demonstrates no significant improvement in accuracy compared to arterial input functions (AIF), venous blood samples are essential for plasma-to-whole-blood (pWB) correction to achieve quantitative values equivalent to the gold standard of AIF with pWB correction. However, when using only whole blood IDIF (without pWB correction), the values are no longer statistically equivalent but remain similar. Depending on the hypothesis and consideration of this bias, or through correction using a population-based function, the pipeline can be rendered fully non-invasive. Furthermore, employing a population-based function for FDOPA metabolite correction aids in maintaining the non-invasiveness of the method. However, it may not be individualistic, potentially impacting patient cohorts with disturbed dopamine systems. Nevertheless, our study demonstrates that using bolus + constant infusion reduces the bias of FDOPA metabolites over a 60-minute window compared to a simple bolus. Additionally, our simulations indicate that even a 40% increase in FDOPA metabolites does not statistically affect the task-specific changes measured in fPET.

While the peripheral metabolism of 6-[^18^F]FDOPA was suppressed by applying Carbidopa and Entacapone, we cannot rule out effects of other metabolites like 6-FDA. As we did not measure metabolite fractions, we were limited to a literature-based correction rather than an individualized approach. While this adjustment might alter dopamine synthesis values to some degree, the metabolism is further reduced by using a bolus + infusion protocol (Supplementary Fig. 3) and simulations indicate negligible changes.

In sum, we propose a robust and accurate protocol to quantify task-induced [^18^F]FDG metabolic changes and 6-[^18^F]FDOPA dopamine synthesis without the burden of arterial blood sampling. Our protocol utilizes IDIFs extracted from thoracic blood pools, which was validated with the gold standard AIF. Furthermore, combining IDIFs across several blood pools showed an excellent match to the AIF. Additionally, the quantitative values derived from the IDIFs were equivalent to those derived from AIF. While the extraction and modelling of IDIF is possible without any invasive measures, to obtain equivalent quantitative measures, correction for pWB using venous blood samples is necessary. Overall, this protocol provides a promising approach to reduce patient burden and experimental complexity while accurately quantifying acute task-specific changes. The approach can be implemented on any PET scanner and offers potential extensions to numerous additional applications.

### Electronic supplementary material

Below is the link to the electronic supplementary material.

Supplementary Material 1

## Data Availability

Raw data will not be made publicly available due to reasons of data protection. Processed data and custom code can be obtained from the corresponding author with a data-sharing agreement, approved by the departments of legal affairs and data clearing of the Medical University of Vienna.
