# Supplementary material for: Validation of cardiac image-derived input functions for functional PET quantification
Source: Eur J Nucl Med Mol Imaging. 2024 Apr 27;51(9):2625–37. doi: 10.1007/s00259-024-06716-8 (PMC11224076; doi:10.1007/s00259-024-06716-8)
Supplement: Supplementary file 1 — Supplementary Material 1 [file 259_2024_6716_MOESM1_ESM.pdf]

Validation of cardiac image derived input functions for functional PET quantification

Murray Bruce Reed[1,2], Patricia Anna Handschuh[1,2], Clemens Schmidt[1,2], Matej Murgaš[1,2], David Gomola[1,2], Christian Milz[1,2], Sebastian Klug[1,2], Benjamin Eggerstorfer[1,2], Lisa Aichinger[1,2], Godber Mathis Godbersen[1,2], Lukas Nics[3], Tatjana Traub-Weidinger[3], Marcus Hacker[3], Rupert Lanzenberger[1,2#], Andreas Hahn[1,2]

[1]Department of Psychiatry and Psychotherapy, Medical University of Vienna, Austria

[2]Comprehensive Center for Clinical Neurosciences and Mental Health (C3NMH), Medical University of Vienna, Austria

[3]Department of Biomedical Imaging and Image-guided Therapy, Division of Nuclear Medicine, Medical University of Vienna, Vienna, Austria

Supplementary Material

# Correspondence to:
Prof. Rupert Lanzenberger, MD, PD
Email: rupert.lanzenberger@meduniwien.ac.at
ORCID: https://orcid.org/0000-0003-4641-9539
Medical University of Vienna, Department of Psychiatry and Psychotherapy, Austria

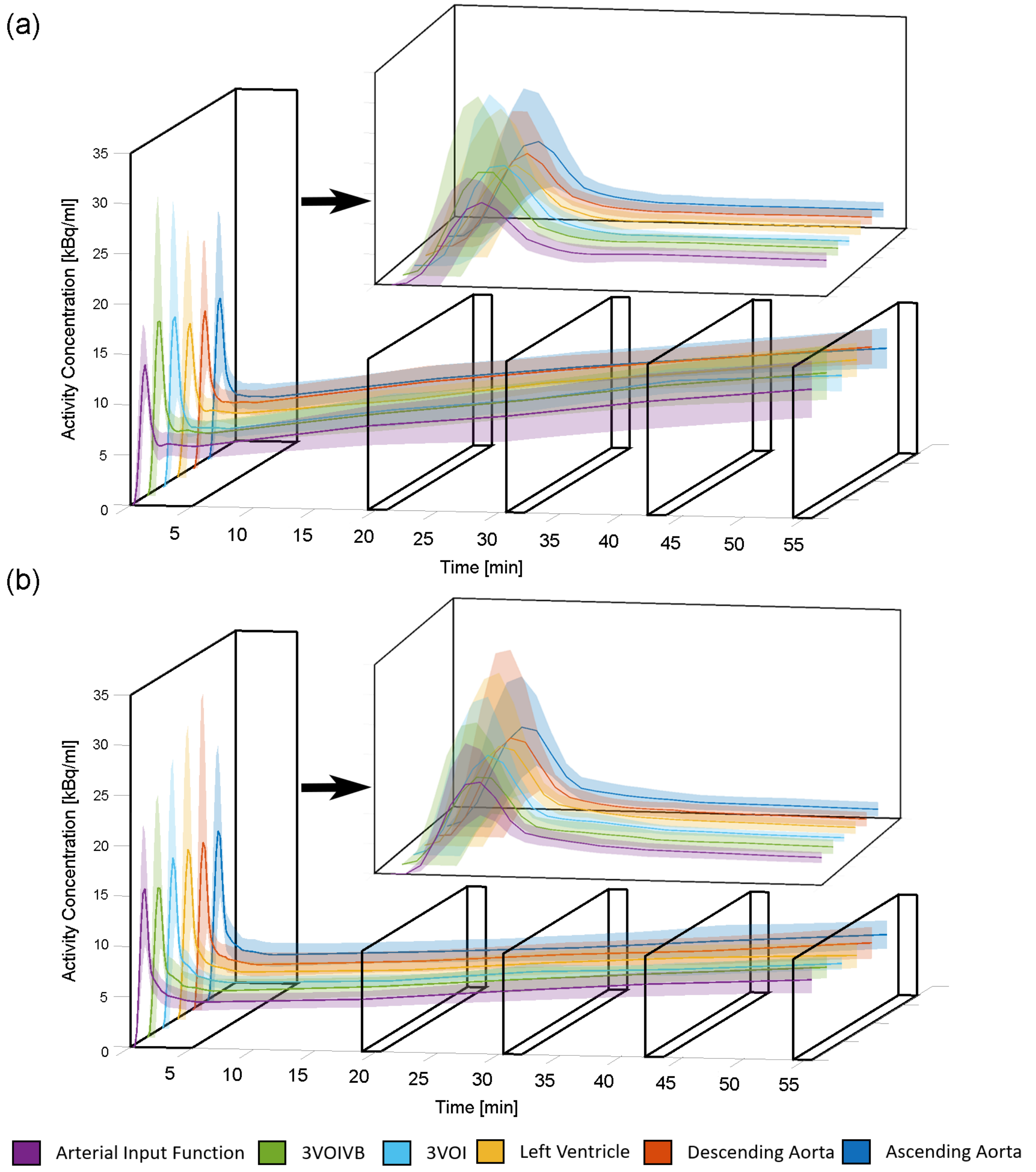

Supplementary Figure 1: Graphical overview of all input functions extracted using the larger VOIs. The mean and standard deviation of each input function's time course for the [^{18}F]FDG (a) and 6-[^{18}F]FDOPA cohort (b). The top right inlay represents the first 5 minutes of the entire time course. The boxes indicate the times when the FOV was shuttled from the brain to the thorax to record the image-derived input function. All input functions display a similar peak. The arterial input function (AIF) peaks later than all image-derived input functions. Finally, both the 3VOI and 3VOIVB display the highest similarity to the AIF.

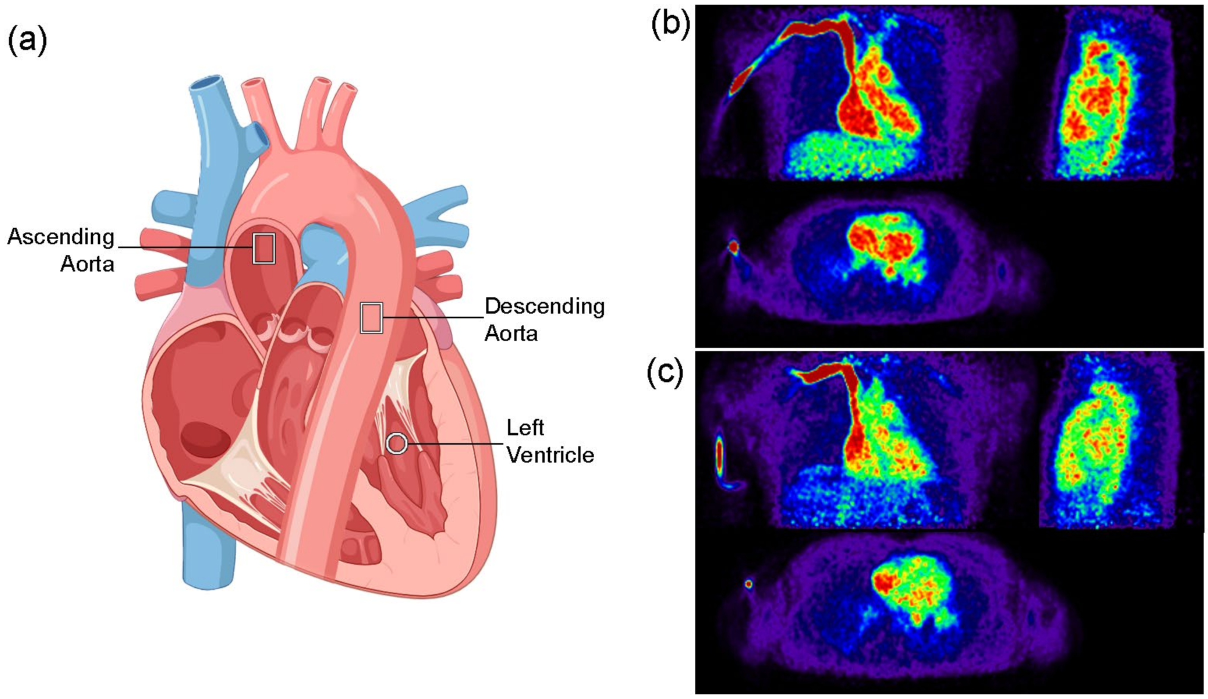

Supplementary Figure 2: (a) Illustrates the intended anatomical positioning of each volume of interest for extracting each image-derived input function. Here the center point was selected and the volume of interest was extracted programmatically. (b) Displays the summed PET thorax image of a single participant in the [^{18}F]FDG cohort, while (c) exhibits the summed image of a participant in the 6-[^{18}F]FDOPA cohort.

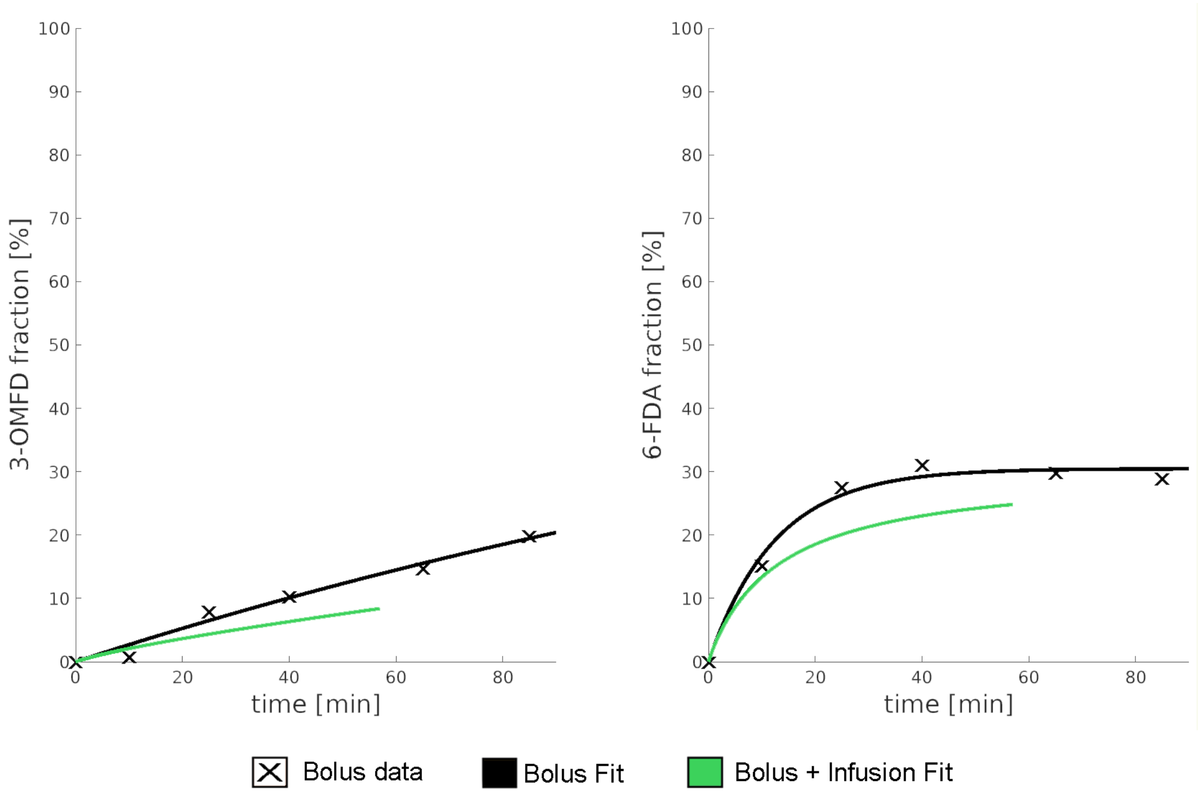

Supplementary Figure 3: The metabolite fractions (3-OMFD) and (6-FDA) were extracted from previous studies [1,2] and [3], respectively (black crosses). These data points were then fitted with a single exponential function (black line) and adapted to match the bolus + infusion protocol (green line), showing the reduced metabolism with this protocol. The estimated bolus + infusion 6-FDA fraction data was combined with the individually measured plasma-to-whole-blood ratio to obtain the corrected arterial and image derived input functions. No corrections for the 3-OMFD fraction were done as participants received 150mg Carbidopa and 400mg Entacapone approximately 1 hour prior to tracer application to block the majority of the peripheral metabolism of 6-[^{18}F]FDOPA. Population based data extracted from [3].

Tracer			[18F]FDG					[18F]FDOPA				
Input Function			Asc Aorta	Desc Aorta	LV	3VOI	3VOIVB	Asc Aorta	Desc Aorta	LV	3VOI	3VOIVB
Brain Regions	Accumbens	r	0.952	0.988	0.989	0.995	**0.997**	0.706	0.969	0.955	**0.968**	0.955
		Slope	2.22	1.11	1.38	1.03	**0.99**	0.74	1.17	1.01	1.06	1.13
		Intercept	-2.14	0.15	-0.44	0.30	**0.26**	<0.00	<0.00	<0.00	<0.00	<0.00
	Caudate	r	0.913	0.982	0.975	0.991	**0.995**	0.734	0.964	0.950	**0.964**	0.948
		Slope	1.67	1.17	1.30	1.10	**1.05**	0.79	1.17	1.01	1.00	1.07
		Intercept	-0.18	0.01	-0.05	0.02	**0.02**	<0.00	<0.00	<0.00	<0.00	<0.00
	Putamen	r	0.865	0.952	0.946	0.977	**0.987**	0.846	0.974	0.971	**0.977**	0.968
		Slope	2.05	1.16	1.39	1.06	**1.03**	0.85	1.17	1.03	1.01	1.07
		Intercept	-0.25	-0.01	-0.07	0.03	**0.02**	<0.00	<0.00	<0.00	<0.00	<0.00
	Oldham*	r	0.975	0.985	0.988	0.995	**0.997**	-	-	-	-	-
		Slope	1.44	1.11	1.19	1.08	**1.06**	-	-	-	-	-
		Intercept	-0.23	0.06	-0.03	0.07	**0.03**	-	-	-	-	-

Supplementary Table 1: Pearson correlation and regression analysis of task-induced [^{18}F]FDG CMRGlu and 6-[^{18}F]FDOPA Ki over all participants between the larger image-derived and arterial input functions. *Cortical regions extracted from the Oldham meta-analysis of the monetary incentive delay task [4]. Bold values represent the best correlation for each tracer and region.

Tracer	Input Function	Brain Regions				
		Cohen's d	Caudate	Putamen	Accumbens	Oldham*
[18F]FDG	Asc Aorta	0.19	0.369	0.097	0.084	0.219
	Desc Aorta	0.18	0.089	<0.009	<0.002	0.091
	Left Ventricle	0.114	0.407	0.023	<0.007	0.131
	3VOIF	0.151	0.025	<0.001	<0.000	0.021
	3VOIFVB	0.930	0.012	<0.003	<0.000	0.040
[18F]FDOPA	Asc Aorta	0.55	0.303	0.140	0.104	N/A
	Desc Aorta	0.54	0.064	<0.002	<0.001	N/A
	Left Ventricle	0.59	0.045	0.060	0.014	N/A
	3VOIF	0.690	<0.003	<0.001	<0.000	N/A
	3VOIFVB	0.480	<0.000	<0.000	<0.000	N/A

Supplementary Table 2: Overview of equivalency test results estimated for regional task-induced net influx rates (Ki) quantified using each image derived-input function extracted from the larger VOIs and arterial input function (significance indicates equivalence). Cohen's d represents the standardized mean difference between the AIF and each IDIF. *Cortical regions extracted from the Oldham meta-analysis of the monetary incentive delay task [4].

Tracer	Input Function	Cohen's d	Brain Regions							
			Caudate		Putamen		Accumbens		Oldham*	
			p-value(1)	p-value(2)	p-value(1)	p-value(2)	p-value(1)	p-value(2)	p-value(1)	p-value(2)
**[18F]FDG**	Asc Aorta	0.19	0.22	0.05	0.19	<0.00	0.16	<0.00	0.07	0.17
	Desc Aorta	0.18	0.04	<0.00	0.01	<0.00	<0.00	<0.00	0.39	0.37
	Left Ventricle	0.11	0.04	0.01	0.05	<0.00	0.02	<0.00	0.26	0.31
	3VOIF	0.33	<0.00	<0.00	<0.00	<0.00	<0.00	<0.00	0.09	0.06
	3VOIFVB	0.22	<0.00	<0.00	0.01	<0.00	<0.00	<0.00	0.12	0.09
**[18F]FDOPA**	Asc Aorta	0.55	0.09	0.23	0.47	0.41	0.46	0.36	N/A	N/A
	Desc Aorta	0.54	0.08	0.04	<0.00	<0.00	<0.00	<0.00	N/A	N/A
	Left Ventricle	0.59	0.33	0.31	0.01	<0.00	0.01	<0.00	N/A	N/A
	3VOIF	0.69	0.30	0.27	<0.00	<0.00	<0.00	<0.00	N/A	N/A
	3VOIFVB	0.05	0.26	0.21	<0.00	<0.00	<0.00	<0.00	N/A	N/A

Supplementary Table 1: Overview of equivalency test results estimated for task-specific CMRGlu ([^{18}F]FDG) or regional net influx rates (Ki, 6-[^{18}F]FDOPA) quantified using each image derived-input function and arterial input function (significance of both p-value(1) and p-value(2) indicates equivalence). Cohen's d represents the standardized mean difference between the AIF and each IDIF. The p-value(1) and p-value(2) represent the left and right one-sided t-test significance values from the two one-sided t-tests (TOST) for each region, respectively. Bold IDIFs show equivalence for all regions of interest. *Cortical regions extracted from the Oldham meta-analysis of the monetary incentive delay task [4].

References

1. Doudet DJ, Chan GL, Jivan S, et al. Evaluation of dopaminergic presynaptic integrity: 6-[18F]fluoro-L-dopa versus 6-[18F]fluoro-L-m-tyrosine. *J Cereb Blood Flow Metab*. Mar 1999;19(3):278-87. doi:10.1097/00004647-199903000-00006

2. Huang SC, Yu DC, Barrio JR, et al. Kinetics and modeling of L-6-[18F]fluoro-dopa in human positron emission tomographic studies. *J Cereb Blood Flow Metab*. Nov 1991;11(6):898-913. doi:10.1038/jcbfm.1991.155

3. Ishikawa T, Dhawan V, Chaly T, et al. Fluorodopa positron emission tomography with an inhibitor of catechol-O-methyltransferase: effect of the plasma 3-O-methyldopa fraction on data analysis. *J Cereb Blood Flow Metab*. Sep 1996;16(5):854-63. doi:10.1097/00004647-199609000-00010

4. Oldham S, Murawski C, Fornito A, Youssef G, Yücel M, Lorenzetti V. The anticipation and outcome phases of reward and loss processing: A neuroimaging meta-analysis of the monetary incentive delay task. *Hum Brain Mapp*. Aug 2018;39(8):3398-3418. doi:10.1002/hbm.24184
